# Regionalization of the antiviral response in the gastrointestinal tract to provide spatially controlled host/pathogen interactions

**DOI:** 10.1128/mbio.02791-22

**Published:** 2023-06-01

**Authors:** Megan L. Stanifer, Stephanie M. Karst, Steeve Boulant

**Affiliations:** 1 Department of Molecular Genetics and Microbiology, College of Medicine, University of Florida, Gainesville, Florida, USA; Ohio State University, Columbus, Ohio, USA; Ohio State University, Columbus, Ohio, USA

**Keywords:** intestinal epithelial cells, interferon, enteric viruses, pattern recognition receptors, compartmentalized immune response

## Abstract

As the largest mucosal surface, the gastrointestinal (GI) tract plays a key role in protecting the host against pathogen infections. It is a first line of defense against enteric viruses and must act to control infection while remaining tolerant to the high commensal bacteria load found within the GI tract. The GI tract can be divided into six main sections (stomach, duodenum, jejunum, ileum, colon, and rectum), and enteric pathogens have evolved to infect distinct parts of the GI tract. The intestinal epithelial cells (IECs) lining the GI tract are immune competent and can counteract these infections through their intrinsic immune response. Type I and type III interferons (IFNs) are antiviral cytokines that play a key role in protecting IECs against viruses with the type III IFN being the most important. Recent work has shown that IECs derived from the different sections of the GI tract display a unique expression of pattern recognition receptors used to fight pathogen infections. Additionally, it was also shown that these cells show a section-specific response to enteric viruses. This mini-review will discuss the molecular strategies used by IECs to detect and combat enteric viruses highlighting the differences existing along the entero-caudal axis of the GI tract. We will provide a perspective on how these spatially controlled mechanisms may influence virus tropism and discuss how the intestinal micro-environment may further shape the response of IECs to virus infections.

The epithelium of the gastrointestinal (GI) tract, being the largest mucosal surface in the body, is constantly exposed to external challenges from both the presence of the commensal microbiota and from various enteric pathogens. Approximately 10^14^ anaerobic bacterial cells are present within the gut lumen, which shape the nutritional network, the physiological environment, and the immune response of the GI tract (1). The architectural features of the intestinal epithelium are unique and consist of repeating invaginations termed crypts of Lieberkuhn, which include the stem cell niche at the base, and are responsible for the continuous renewal of the epithelial monolayer of the gut (Fig. 1) (2). Stem cells in the crypts cycle slowly and divide asynchronously to give rise to a daughter stem cell and a transient amplifying (TA) cell (3). The TA cells are fast cycling cells that differentiate and migrate upward toward the tip of protrusions reaching into the intestinal lumen, called villi (3). There are TA cells that lead to both the absorptive and secretory cell lineages found in the intestine, and these cells differ in morphology and carry out specialized functions (Fig. 1A) (4). Enterocytes represent the most common cell type within the intestinal epithelium (80% of the cells) and drive the digestive enzyme production and nutrient absorption (5). Goblet cells are secretory cells that produce the components of the mucus matrix. The secretory Paneth cells have two critical functions: first is to produce anti-microbial compounds that in turn regulate the composition of the intestinal flora (6) while its second function is to support the stem cell niche. Importantly, Paneth cells are only found in the small intestine and are absent in the colon (Fig. 1A). Enteroendocrine cells also belong to the secretory cells and generate neuropeptides and hormones to enable communication with the enteric nervous system (7). As part of a continuous turnover of the epithelial monolayer, fully differentiated cells are shed into the gut lumen, and the entire intestinal tract is turned over every 3–5 days (1, 2).

**Fig 1:**
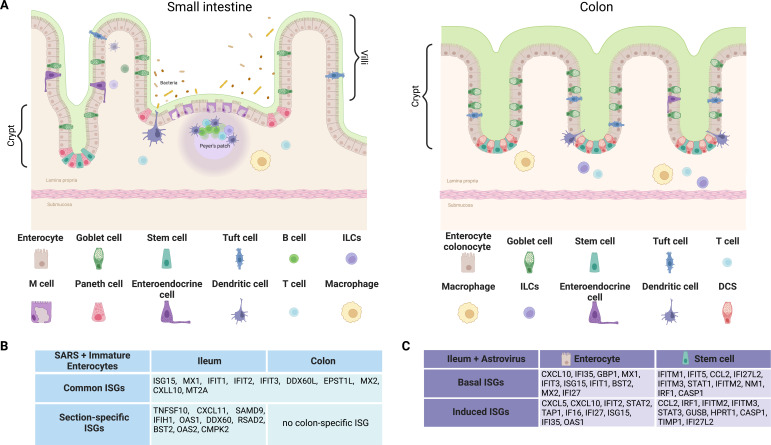
Cell-type-specific response of intestinal epithelial cells. (**A**) Schematic depicting the small intestine and colon displaying the cell structure, the location of each intestinal cell types, and the mucus levels found in these two sections. Image was made in Biorender. (Innate lymphoid cells (ILCs), deep crypt secretory cells (DCS)) (**B**) Section-specific ISGs found in immature enterocytes following SARS-CoV-2 infection of human ileum and colon organoids described by Triana et al. (8). These data were derived from single-cell sequencing of human colon and ileum organoids from the same patient donor. (**C**) Cell-type-specific ISGs found following human astrovirus 1 infection of human ileum organoids described by Triana et al. (9). These data were derived from single-cell sequencing of human ileum organoids from a single donor.

The GI tract itself can be divided into six regions starting from the stomach, duodenum, jejunum, ileum, colon, and rectum (Fig. 2A and B). These regions are each distinct in terms of their cell compositions (e.g., the lack of Paneth cells in the colon), absorptive properties, the length of their villi (e.g., the villi are longer in the duodenum and jejunum compared with ileum and are absent in the colon), their type and quantity of mucus, and their diversity and quantity of commensals (Fig. 1A) (10). In this manuscript, we will review an important emerging concept that each GI section responds uniquely to enteric viruses. We will discuss how this compartmentalization of the host response to enteric pathogens could be important for virus tropism and for maintenance of gut homeostasis; however, we will focus on the small and large intestine and not discuss the stomach or rectum.

**Fig 2:**
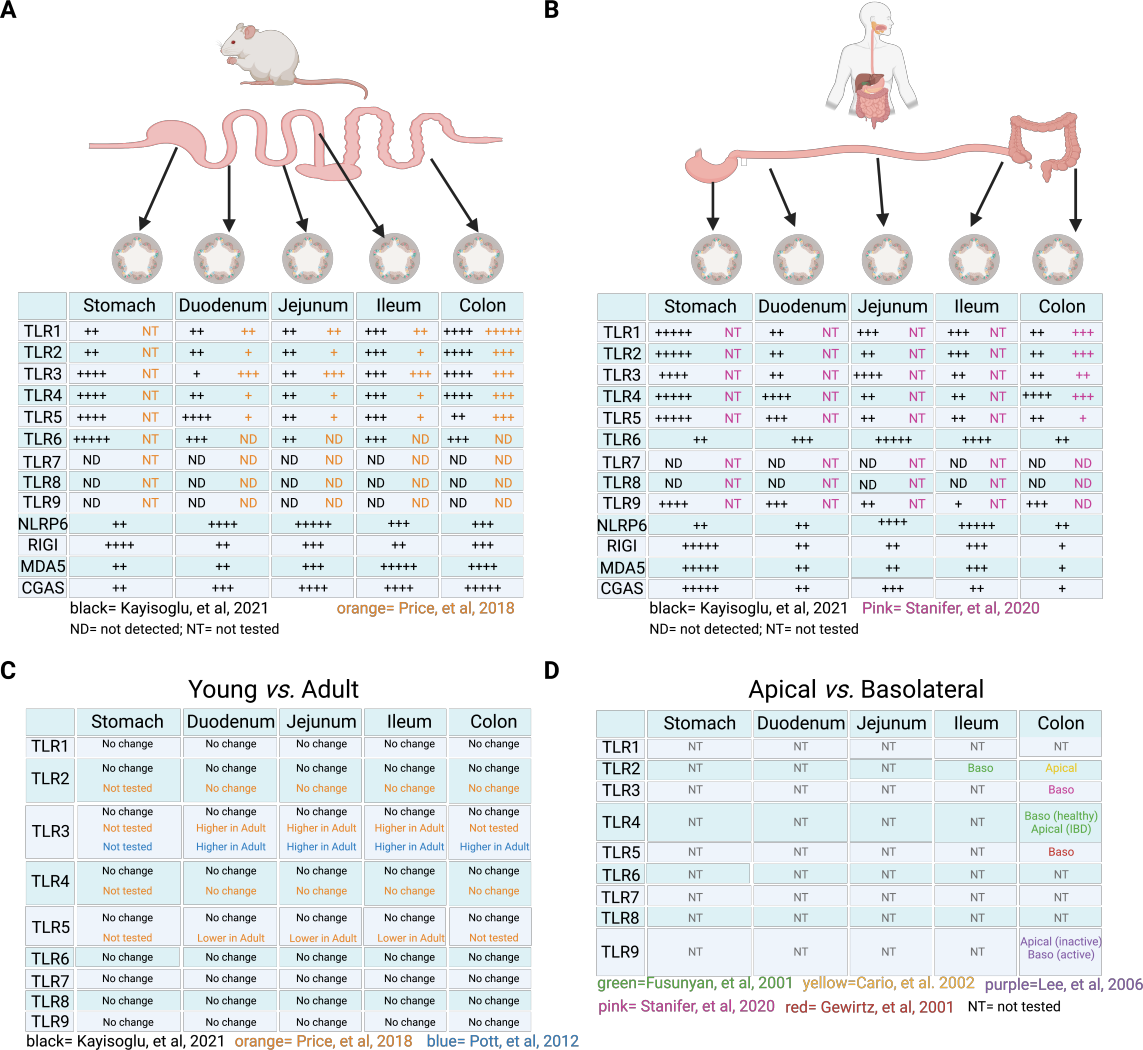
Section-specific localization of PRRs. (**A**) Diagram showing the murine gastrointestinal tract and the PRRs which have been shown to be present by RNA sequencing by Kayisoglu et al. (10) (black, from C5BL/6 mice, from the data presented in Fig. 2 of the paper, −4, +; −2, ++; 0, +++; 2, ++++; 4, +++++) and from qPCR and RNA sequencing by Price et al. (11) (orange, from C5BL/6 mice). (**B**) Diagram showing the human GI tract and the PRRs which have been shown to be present by RNA sequencing by Kayisoglu et al. (10) (black, derived three independent human donors for each section, from the data presented in Fig. 2 of the paper, −4, +; −2, ++; 0, +++; 2, ++++; 4, +++++) and from qPCR sequencing by Stanifer et al. (12) (pink, T84 cells and human colon organoids from three donors). (**C**). The expression of TLRs1–9 was compared from RNA sequencing from murine samples by Kayisoglu et al. (10) (black, from C5BL/6 mice), qPCR by Price et al. (11) (orange, from C5BL/6 mice), and from qPCR by Pott et al. (13) (blue, from C5BL/6 mice). (**D**). Apical and basolateral distribution of TLRs1–9 in human IECs as determined from antibody staining of the receptors. Green, Funsunyan et al. (14) (from human ileum cells); yellow, Cario et al. (15) (from T84 cells); pink, Stanifer et al. (12) (from T84 and human colon organoids); red, Gewirtz et al. (16) (from T84 cells); purple, Lee et al. (17) (from HT-29 cells). ND, not detected; NT, not tested.

## PATHOGEN SENSING BY INTESTINAL EPITHELIAL CELLS

The cells of the intestinal epithelium form a barrier to separate the external environment from the underlying tissues, protecting the body against pathogen entry. Intestinal epithelial cells (IECs) are a primary target of enteric viruses, including rotavirus, orthoreovirus, picornaviruses, norovirus, coronaviruses, and astrovirus. Enteric virus infection can result in acute GI distress by causing nausea, vomiting, diarrhea, and fever and can be associated with long-term post-infectious disorders such as irritable bowel syndrome and celiac disease (18). The number of known enteric viruses is still increasing due to modern sequencing methods aiming to provide a better knowledge of the virome in the human intestinal tract.

Viruses enter the GI tract following ingestion of contaminated food or water. These enteric viruses can infect the intestinal epithelium by either attaching and entering from the apical membrane, being endocytosed and released from either apical or basolateral endosomes, by transiting through M cells to infect the basolateral membrane of IECs (19) or by using transepithelial dendrites or goblet cell-associated antigen passages to pass from the lumen into the lamina propria (Fig. 1A) (20, 21). Following virus entry and replication of the viral genomes, IECs mount an intrinsic innate immune response against the invading pathogens. This immune response results in the production and secretion of pro-inflammatory cytokines and type I and type III interferons (IFNs) (22). IFNs then bind their cognate receptors on the epithelial cells to induce IFN-stimulated genes (ISGs), which act to control virus infection and mitigate systemic infection of the body (22). While numerous immune cells localize under the intestinal epithelium and participate in the overall host response to enteric pathogens in a regional manner (Fig. 1A) (23), this review will strictly focus on the response of the epithelial cells present along the GI tract to virus infection.

## PATTERN RECOGNITION RECEPTORS ARE DIFFERENTIALLY EXPRESSED ALONG THE GI TRACT

IECs play a key role in maintaining and fine-tuning intestinal homeostasis, where they must be responsive against invading pathogens but must not overreact and cause excessive inflammation (24). To achieve this balanced response, IECs have developed key strategies to compartmentalize their detection of pathogens. They express a wide variety of pathogen recognition receptors (PRRs) such as Toll-like receptors (TLRs), Retinoic acid-inducible gene I (RIG-I)-like receptors (RLRs), and Nucleotide-binding and oligomerization domain (NOD)-like receptors (NLRs). These PRRs recognize pathogen-associated molecular patterns (PAMPs) that comprise molecular structures shared by different microorganisms (25). PAMPs of viruses are usually nucleic acids which can be the viral genome itself (25) or specific nucleic acid intermediates that are created during virus replication (e.g., double-stranded RNA structures during RNA virus replication). Viral nucleic acids, including single-stranded (ss)RNA and double-stranded (ds)RNA, are sensed by RNA sensors such as TLR3 and TLRs7–9, RLRs (RIG-I and melanoma differentiation-associated gene 5 (MDA-5), or in case of viral DNA by DNA sensors such as absent in melanoma 2 and cyclic GMP–AMP synthetase (cGAS) and stimulator of interferon genes (STING) (25, 26). These PRRs sense foreign nucleic acids in endosomal compartments (e.g., TLRs) or in the cytosol (e.g., RLRs and NLRs) of the cell (27). Upon PAMP detection, the PRRs recruit adaptor molecules to elicit a signaling cascade through either the NFκB pathway or the IRF3/IRF7 pathway (25 - 27). The induction of these pathways leads to expression and secretion of pro-inflammatory cytokines or type I and type III IFNs, respectively (25, 27). Secreted cytokines bind their cognate receptors on the infected cell (autocrine) or on neighboring uninfected cells (paracrine) to help protect the epithelial monolayer. This coordinated sensing of pathogens is key to control the barrier integrity and prevent systemic infections.

A recent study has evaluated the presence of the key PRRs which recognize and control virus infection (RLRs, TLRs, and cGAS) along the murine and human GI tract. This report used organoids from three donors derived from the stomach, duodenum, jejunum, ileum, and colon (10). Following RNA sequencing of these organoids, the authors found that TLR3 and RIG-I are mostly expressed in the stomach, while MDA-5 and cGAS are mainly expressed in the distal small intestine and colon in murine models (Fig. 2A). Interestingly, humans displayed a distinct distribution with RIG-I, MDA-5, and cGAS mostly being expressed in the stomach (Fig. 2B). TLR7 was not found to be expressed in any section for both murine and human models (Fig. 2A and B). Interestingly, TLR9 was also absent in murine IECs but was found to be lowly expressed in all parts of the small intestine and colon in humans except for one donor which showed high expression in the colon (Fig. 2A and B) (10). Work from our lab has confirmed these findings and shown that human colon organoids only elicit an immune response following stimulation by TLR1-5 ligands, and human colon organoids do not elicit an immune response to TLR7 and TLR9 stimuli (Fig. 2B) (12). These spatially distributed PRRs pose an interesting question on where and how viruses can be detected; however, many studies only provided limited functional assays to show whether these distinct PRR expression patterns are linked to restricted responsiveness to their respective stimuli.

Viral PRRs are not the only ones that have been shown to have a section-specific distribution in the murine and human GI tract (7, 10, 11, 28). TLRs that recognize bacteria and NLRs have also been highly studied and shown to have section-specific localizations (10). TLR1-6 can be expressed by human and murine IECs. RNA sequencing of human and murine organoids from six intestinal sections revealed that murine gastric sections preferentially express TLRs3–6, small intestinal sections express low levels of all TLRs, and the colon expresses high amounts of TLRs1–4 (10) (Fig. 2A). Interestingly, human TLRs are distributed in a slightly different manner with gastric sections expressing high levels of TLRs1–5, small intestinal sections expressing high levels of TLR6, and the colon not expressing high levels of any TLRs (Fig. 2B) (10). As the murine samples used in this analysis were genetically similar (C57BL/6 strains), their TLR expression was highly conserved between mice. On the contrary, human samples showed a larger variation in the expression of different TLRs. Genetic variability between samples might explain these differences, suggesting that genetic differences between individuals could influence how TLRs are distributed along the GI tract (10). An alternative explanation for the differences observed between human samples could be that the patients had nonsymptomatic underlying inflammation or infection at the time of sample collection, impacting TLR expression in the respective patient-derived organoids.

The regional compartmentalization of TLRs was confirmed using TLR reporter mice, which were engineered to express TLRs2, 4, 5, 7, and 9 containing an HA or a FLAG tag with the concurrent expression of a fluorescent protein (11). This study also showed that TLR7 and TLR9 are not expressed in the IECs themselves, but they are found in the underlying lamina propria (Fig. 2A) (11). Using *in situ* hybridization, the authors found that the murine small intestine had a very low expression of TLRs2 and 4, while TLR5 was expressed at high levels only in Paneth cells residing in the stem cell crypts (Fig. 2A) (11). The proximal colon showed high levels of TLRs2, 4, and 5, while the distal colon only showed a high expression of TLR2 (Fig. 2A) (11).

Importantly, several groups have demonstrated that this unique TLR compartmentalization could be fully dependent on the identity of the tissue section itself and is not influenced by outside factors such as microbiota or immune cells. Price et al*.* showed that neonatal and adult mice have the same distribution of TLRs2, 4, and 5 in both the small intestine and the colon (Fig. 2C) (11). Interestingly, they found that TLR5 expression was similar in the colon but that neonatal mice had high levels of TLR5 in their small intestine which diminished as the mice aged and became solely localized in Paneth cells in adult animals (Fig. 2C) (11). The authors further showed that this change in localization was not dependent on TLR5 signaling or signals from commensal microbiota as mice lacking the TLR5 signaling adaptors or germ-free and special pathogen-free (SPF) mice all displayed the same TLR5 distribution pattern as seen in wild-type animals (11). Kayisoglu et al*.* confirmed these results by creating murine intestinal organoids from embryonic mice (10). Since embryonic mice are maintained in a sterile environment and have not yet been exposed to microbiota, their intestinal TLR patterning will be unaffected by the presence of the microbiome. Interestingly, Kayisoglu et al. used RNA sequencing to show that embryonic expression patterns of TLRs corroborated the expression patterns seen in adult mice (Fig. 2C) (10). Furthermore, they could show that these embryonic organoids were responsive to external TLR4 stimulation in a compartmentalized pattern similar to adult organoids (e.g., stomach but not small intestinal organoids was responsive to TLR4 stimulation by LPS) (10). On the other hand, TLR3 expression was found to increase from neonatal to adult mice (Fig. 2C) (11, 13). Sequencing from human tissue samples also confirmed that TLR3 levels increase in adults (13). This increase in TLR3 expression correlated with reduced susceptibility of mice to rotavirus infection (13).

Taken together, these studies have revealed that all PRRs are expressed in distinct sections of the GI tract; however, more work needs to be done to functionally challenge many of these PRRs to determine if their expression correlates with their ability to sense and respond to pathogens. Additionally, many of these studies looked at RNA expression but did not correlate how this relates to protein expression in the specific sections. Importantly, these studies also showed that this compartmentalization is predetermined at birth for most PRRs, and a few TLRs (TLR3 and TLR5) have expression levels that change with the age; however, the molecular determinants for this change in expression remain unclear.

## INTRACELLULAR COMPARTMENTALIZATION OF TLRs IN IECs

In addition to section-specific localization of TLRs, the intracellular localization of TLRs has also been explored. IECs are polarized cells with an apical side facing the lumen of the intestine and a basolateral side facing the lamina propria. The establishment of this polarization requires finely tuned protein trafficking and sorting, which allow for the apical and basolateral membranes to have distinct protein and lipid compositions (29). Several TLRs have been found to have polarized protein localization in IECs. TLR5, which detects flagellin, has been shown to be present solely on the basolateral membrane of human colon carcinoma cells (Fig. 2D) (16). This basolateral localization of TLR5 restricts sensing to bacteria that have breached the intestinal lining and thus avoiding the elicitation of inflammation to commensal bacteria present in the gut lumen (16). TLR3, the main sensor of dsRNA, has also been shown to be localized to the basolateral side of both colon carcinoma cell lines and human colon organoids (Fig. 2D) (12). TLR3 is trafficked to the basolateral side of IECs by the clathrin-sorting adaptor AP1 (12). As for TLR5 sensing of enteric bacteria, this basolateral localization of TLR3 restricts its sensing of viral PAMPs to infections initiated at the basal surface when an enteric virus has breached the epithelium. Importantly, patients with inflammatory bowel disorders have been shown to have defects in AP1, raising the possibility that its loss leads to uncontrolled pathogen sensing (30). Interestingly, some TLR proteins are localized to both apical and basolateral membranes, but they are only capable of signaling from one membrane. For example, while TLR9 is expressed at the apical and basolateral surface of human colon carcinoma cells and can bind agonist on both membranes, signaling from basolateral stimulation leads to a strong activation of NF-kB through canonical pathways, whereas apical stimulation leads to accumulation of ubiquitinated IkappaB, thereby leading to shunted NF-kB signaling (Fig. 2D) (17). The localization of TLRs2 and 4 is also polarized in many cells, but this can change depending on the section of the GI tract. For example, TLR2 is basolateral in human fetal ileum tissue (14) but apical in polarized colonic T84 cells (Fig. 2D) (15). Murine TLR2 is apical in the villi but both apical and basolateral in follicle-associated epithelial cells overlying Peyer’s patches (31). TLR4 is basolateral in the colon of healthy human patients but has an increased expression and an apical localization in patients with ulcerative colitis or Crohn’s disease (Fig. 2D) (14). These sections of species-specific differences in TLR localization have only been evaluated for handful of TLRs and require further studies to fully understand how other TLR localizations and activities are regulated (11, 32).

TLRs are not the only PRRs that have been shown to have an intracellular compartmentalized response. The RLR adaptor MAVS has been shown to elicit distinct signaling pathways depending on whether MAVS signals are from the mitochondria, the peroxisome, or mitochondria-associated membranes (MAMs) (33, 34). Peroxisome-activated MAVS triggers type III IFN expression, whereas mitochondria-activated MAVS triggers type I IFN expression. Type III IFNs are key antiviral cytokines for epithelial cells (22). Importantly, IECs have a large pool of peroxisomes which could explain why these cells preferentially produce type III IFNs in contrast to most other cell types which produce abundant type I IFNs (35). MAM provides a link between the endoplasmic reticulum (ER) and the mitochondria and peroxisome (34). Interestingly, it has been suggested that MAM-associated MAVS coordinates mitochondrial and peroxisomal MAVS signaling. MAM disruption prevents mitochondria-associated MAVS signaling, shifting signaling to the peroxisomal fraction (34). Taken together, these results suggest that the site of RLR signaling can dictate the type of intrinsic immune response generated upon virus infection.

## COMPARTMENTALIZATION OF IFN RECEPTORS ON EPITHELIAL CELLS

IECs protect themselves against pathogen infection by upregulating type I and type III IFNs. Type I IFNs signal through the ubiquitously expressed heterodimeric receptor complex consisting of IFNAR1 and IFNAR2, while type III IFNs signal through the epithelial-specific IFNLR1 and the ubiquitous IL10-Rb receptors. Following binding of the IFNs to their receptors, a JAK/STAT signaling cascade is elicited, which leads to the upregulation of hundreds of ISGs that act to control the spread of the pathogen.

Over recent years, several studies have examined the nature of the IFN response responsible for controlling specific virus infections in the intestinal tract. Type III IFNs play an important role in controlling rotavirus, reovirus, norovirus, and SARS-CoV-2 infections of IECs in both human and murine hosts (36 - 42). While type I IFNs protect against intestinal immune cell infection and systemic spread of enteric viruses, they are less important for protecting murine IECs from several viruses including reovirus, norovirus, and adult animals infected with rotavirus (38 - 42). This epithelial specificity for type III IFNs is due to the distinct expression of the type III IFN receptor subunit, IFNLR1, mostly on epithelial cells, while the type I IFN receptor subunit, IFNAR1, is found exclusively on lamina propria cells in adult mice and not on epithelial cells (39). This restricted expression leads to IECs being more responsive to type III IFNs and inducing higher amounts of ISGs, such as Mx1, while lamina propria cells induce high ISGs in response to type I IFN stimulation (38). It has also been shown that type I IFNs induce inflammatory phenotypes which would be detrimental to epithelial cells (43, 44). Interestingly, the lack of IFNAR1 responsiveness on IECs was found to be age dependent as IECs from adult mice were unable to induce pSTAT1 after type I IFN stimulation while IECs from neonatal mice respond to type I IFN treatment (44, 45). Additionally, type I IFNs were important to control persistent infection of murine astrovirus (46) and could control reovirus, rotavirus, and norovirus infection of human IECs (42, 47 - 49). Furthermore, when murine or human epithelial cells are treated with type I IFN *ex vivo*, they are able to elicit an immune response (38, 43, 44, 47, 50, 51), suggesting that the intestinal environment (such as the microbiota or immune cells) downregulates the expression of the type I IFN receptor or inhibits its signaling in epithelial cells.

## REGIONALIZATION OF THE IFN RESPONSE DETERMINES ENTERIC VIRUS TROPISM

Just as PRRs are not uniformly expressed along the intestinal tract, virus infection does not occur in a uniform manner. Enteric viruses display a preference for certain regions of the GI tract, determined by both host and microbial factors. Moreover, there is a regulation of viral infections along the crypt-villus axis. We will describe examples of both types of regionalization in this section.

Murine norovirus (MNV) strains display variable preferences for different regions of the GI tract. For example, MNV1 preferentially infects the ileum of the small intestine, while CR6 and MNV3 infect the colon (52). While the basis for this virus strain-dependent difference is not fully understood, it is regulated by intestinal microbiota since both ileum-targeted MNV1 and colon-targeted MNV3 shift to infecting the duodenum in antibiotic-treated mice depleted of intestinal microbes (52). These data imply that commensal bacteria normally trigger a protective response in duodenal tissue that prevents infection by multiple MNV strains. We have shown that this response requires type III IFNs and that bacterial depletion can be rescued by supplementing mice with bacterially derived bile acids, establishing a model whereby commensal bacteria generate certain classes of bile acids that prime IFN responses in the duodenum (52). Our work and that of other groups confirm that bile acids can augment IFN induction and play key antiviral roles *in vivo* (52 - 54). The regional nature of this immune response remains unresolved, but a possible explanation is that there are regional differences in expression of a bile acid receptor(s) that primes IFN induction. The shift of MNV infection from the ileum (for MNV1) or colon (for MNV3 and CR6) to the duodenum in antibiotic-treated mice also implies that intestinal microbiota are responsible for determining MNV tissue tropism. While it is unclear what drives variable tropism among MNV strains, environmental cues, including bile acids and pH, cause MNV virion contraction, enhancing interaction with the host receptor CD300lf, so it is possible that differential sensitivity to these microbiota-dependent environmental cues could contribute. Supporting this possibility, we recently uncovered MNV strain-dependent differences in sensitivity to these environmental factors, with ileum-tropic viruses being more sensitive than colon-tropic ones (55).

Interestingly, rotavirus infection of murine models has been shown to preferentially infect the small intestine in the duodenum, jejunum, and ileum. Use of human small intestinal organoids has also confirmed that human rotavirus can infect and replicate in these three sections of the GI as well (56). This infection of the small intestine displays a further important characteristic whereby the tip of the intestinal villi is preferentially infected by rotavirus (38, 57, 58). In *Ifnlr1^−/^*^−^ and *il22^−/−^* mice, this infection spreads further down the villi toward the crypt region (38, 57). This observation suggests that, within the villus structure, cells located toward the middle of the villus have a higher basal immune response which can restrict virus infection from these sites. On the contrary, cells located toward the tip may have a lower immune response, permitting the higher susceptibility of this region to virus infection. Allowing the tips to be more susceptible can be a strategy by the host to allow for virus infection to establish in this region, as the gut lining is shed every 3–5 days, these vulnerable cells would die soon and be shed regardless of infection, thereby protecting the host from tissue destruction. However, the mechanism used by cells to establish this gradient of immune response along the crypt-villi axis has not been uncovered. Alternatively, the tips may just be the first contact with a virus and lead to infection occurring preferentially at these points. As the mucus layer is often thinner at the tips, this could provide an easier access point for invading viruses. Further work is required to distinguish between these possibilities.

A further method that could drive viral tropism and regional virus infection is the presence of antiviral hotspots. A recent paper from Van Winkle et al*.* has demonstrated that under homeostatic conditions, murine IECs contain areas that express high amounts of ISGs (59). These ISG hotspots are regions of the epithelium where a patch of cells displays high levels of ISGs. The authors determined that the ISGs were induced in these patches due to the sensing by IECs of type III IFNs derived from epithelium-associated CD45+ leukocytes (59). Mice which lack the type III IFN receptor do not display these hotspots and are more susceptible to virus infection due to the loss of this regional protection. Analysis of single-cell RNA sequencing of healthy patient ileum samples (60) reveals that mature enterocytes in humans also display a greater ISG expression compared with less differentiated cells that are found in the crypt region (59). This suggests that similar antiviral hotspots could also be present in the human GI tract.

## CELL-TYPE-SPECIFIC RESPONSE TO ENTERIC VIRUS INFECTION

A recent study by Moor et al. has used microdissection of the crypt-villi axis to determine how enterocytes mature as they move away from the crypts toward the villi (61). This work dissected the crypt-villi axis into five sections and used single-cell sequencing to show that enterocytes transition through several states during this migration and become multiple distinct cell types along the way (61). This work shows that enterocytes are not terminally differentiated but are rather four to five distinct cell populations (61). Single-cell sequencing of virus-infected human intestinal organoids has confirmed these findings and shows at least four distinct enterocyte populations are present in both noninfected and infected cells (8, 9, 62). These enterocyte populations are often termed immature enterocytes 1 and 2 and mature enterocytes 1 and 2 and have been shown to support virus infection in distinct manners (8, 9). Interestingly, single-cell sequencing of rotavirus-infected mouse models has revealed that rotavirus infection leads to increased cell proliferation and increased cycling stem cells. This increased activity acts as a response to repair the damaged tips following infection suggesting that the population and the number of enterocytes can vary under infection and repair conditions (58). Infection of both human ileum and colon organoids by SARS-CoV-2 has shown that SARS-CoV-2 has a highly restricted cellular tropism whereby it mainly infects immature enterocyte 2 cells in both the ileum and the colon, while immature enterocyte 1 and mature enterocytes do not support SARS-CoV-2 infection (8). Importantly, these results revealed that the preference of SARS-CoV-2 for infecting these cells was not due to high expression of its cellular receptor ACE2, as mature enterocytes expressed the highest amounts of ACE2 but supported the lowest amount of virus infection (8). These results suggest that while a small amount of ACE2 is required for virus attachment and entry, additional factors which are cell type-specific are required to facilitate a productive SARS-CoV-2 infection. Interestingly, these SARS-CoV-2 studies have shown that the ileum and colon respond to virus infection in distinct manners. The immature enterocyte 2 cells supported the highest amount of SARS-CoV-2 infection in both the ileum and colon; however, when evaluating the top 30 upregulated ISGs upon virus infection, immature enterocyte 2 of ileum and colon induced the expression of several common ISGs, but the ileum also upregulated section-specific ISGs not found in the colon (Fig. 1B). These studies were performed in ileum and colon samples from the same patient indicating that this is not a patient-specific pattern but that the same cell type in different intestinal sections will respond to virus infection in a distinct manner. Interestingly, human astrovirus VA1 was used to infect organoids from multiple intestinal sections (duodenum, jejunum, ileum, and colon) from both children and adults. In this work, Kolawole et al. evaluated the upregulation of four ISGs (SG15, MX1, OAS2, and RSAD2) and found that most intestinal sections upregulated these four ISGs but to different magnitudes depending on the donor or the section (63). While this study evaluated the response of the whole organoid and not individual cell types, this suggests that while colon immature enterocytes did not make OAS2 or RSAD2 following SARS-CoV-2 infection they may be able to when infected with a different virus or that different cells in the colon will make OAS2 and RSAD2 in response to astrovirus infection (63). Future work dissecting single-cell responses to multiple enteric viruses will be needed to determine which of these mechanisms are occurring.

Additional single-cell sequencing studies of human intestinal organoids infected by human astrovirus 1 have revealed that each cell type responds to virus infection in a distinct manner. These studies revealed that unlike murine astrovirus (64), human astrovirus 1 displays a broad cellular tropism and infects all cells within the human intestinal tract (9, 63). Analysis of these infected cells showed that while stem cells, TA cells, enteroendocrine, and enterocytes support infection, each cell type upregulated unique antiviral programs. In each of these cells, the type of ISGs used to combat the virus infection was distinct (e.g., stem cells upregulated CCL2, IFI27, and S100P, while enterocytes preferentially upregulated OASL, RSAD2, and CCL5) (Fig. 1C). Importantly, this work showed that, at basal levels, each cell type within the human GI tract displayed their own unique levels of antiviral ISGs (9). These basal levels of ISGs were distinct between all cell types (e.g., in noninfected cells, stem cells expressed high levels of IFITM3, STAT1, and IRF1, and enterocytes expressed high levels of CXCL10, MX1, and IFIT3) (Fig. 1C). While these unique antiviral environments did not impact human astrovirus 1 infection, they could be key in controlling other enteric virus infections leading to more restricted cellular tropisms like that seen in SARS-CoV-2 infected organoids.

## CONCLUSION AND PERSPECTIVES

The maintenance of gut homeostasis is a critical challenge for both the epithelial cells lining our GI tract and for the tissue-resident immune cells located in the lamina propria. The emerging concept is that to achieve homeostasis, expression of PRRs and their downstream signaling must be specifically tailored. On the one hand, IECs need to show a certain level of blindness to tolerate the presence of the commensal flora. However, on the other hand, they need to be responsive against pathogens, and some level of PRR-mediated signaling is important to mediate tissue repair and achieve barrier integrity and homeostasis.

Our understanding of the complexity of the immune response generated by IECs upon enteric virus challenges is only at its infancy. IECs are immune competent and able to elicit strong antiviral responses. As such, they intrinsically play a key role in the first line of defense against enteric viruses. However, whether, like the commensal flora, a finely tuned balance between response and partial blindness to enteric viruses is beneficial to achieve and maintain gut homeostasis remains unknown. This is an important question that should be addressed as deep sequencing methods had revealed the presence of a great number of viruses in the lumen of the gut. This virome, which is mostly present in healthy patients who do not have GI symptoms, is made up of mostly bacteriophages; in addition, there are viruses that infect human cells and other organisms such as archaea as well as viruses that are present in food and are present in a transient manner (65). This suggests that these viruses replicate in an asymptomatic manner in IECs or that they are not able to replicate and are simply the result of our food uptake. Whether the simple presence of this virome can provide flares to our epithelium and participate in regulating gut homeostasis remains unknown, and further studies are required to appreciate whether they act in a stimulatory manner toward IECs or if they are just passive interactors.

Future work is required to further analyze how epithelial cells from the stomach, jejunum, duodenum, ileum, and colon coordinate their immune responses to virus infection. Single-cell sequencing of infected ileum and colon organoids has revealed both section-specific and cell-type-specific responses. It is critical to continue to evaluate these cell-type-specific responses with more enteric pathogens to determine whether they are correlated with specific virus tropism or whether the cell-type-specific ISG expression signatures are the consequence of the primary functions of various intestinal cell lineages cells. Importantly, organoids do not contain microbiota or immune cells which may influence the priming and basal ISG inductions *in vivo* as well as how they respond to pathogens. Additionally, it is key to evaluate environmental factors present in the intestinal tract that can impact virus infection. While a lot of work has been performed in the recent years looking at how the microbiota and immune cells coordinate virus sensing and clearance (66), there has been little work looking at how the low oxygen (hypoxic) environment in the intestine impacts antiviral responses. Hypoxia has been shown to play a key role in barrier integrity (67) as it induces the secretion of trefoil factors and microRNAs, which aid in repairing and maintaining a tight epithelial barrier (67, 68). However, a hypoxic state is created by the expression of key transcription factors, which are also known to modulate immune functions. It will be important to begin to understand whether these changes to immune functions in hypoxia impact how cells fight pathogens and how overall changes to the gut microenvironment impact the ability of cells to maintain homeostasis.

Overall, the IECs lining the GI tract have used several strategies such as the regionalization of PRR expression, regulating the apical or basolateral expression of PRRs, upregulating cell-type-specific ISGs, and interacting with section-specific microbiota to maintain tolerance in the midst of their complex environment. Many future studies using human primary cells and models that better replicate the biochemical and biophysical properties of the gut are required to fully appreciate the intricate communication which achieves homeostasis and controlled immune sensing in this tissue.
